# Benefits and challenges of transitioning preterm infants to at-breast feedings

**DOI:** 10.1186/1746-4358-1-13

**Published:** 2006-08-31

**Authors:** Kathleen M Buckley, Gloria E Charles

**Affiliations:** 1 The Catholic University of America, School of Nursing, Washington, DC, USA; 2 Holy Cross Hospital, Neonatal Intensive Care Unit, Silver Spring, MD, USA

## Abstract

Upon hospital discharge it is not unusual for mothers of preterm infants to continue to meet all or most of their infants' nutritional needs through bottle feedings of expressed breast milk (EBM) because of infants' physiological immaturity and maternal concerns with an inadequacy of milk supply. Although for some mothers the challenge of transitioning the infant to feeding at the breast may be beyond their ability and resources, for others it appears to be based on a conscious choice. Mothers are often unaware of the advantages of breastfeeding at the breast. The purpose of this article is to examine some of the factors that may contribute to the inability and resistance of mothers to transition their preterm infants, and to report on the potential short and long-term advantages associated with feeding at the breast as opposed to feeding bottles of EBM.

## Review

Breast milk for preterm infants has been found to reduce the health risks associated with feeding infant formula including a higher incidence of infections and necrotizing enterocolitis [1,2], lower scores on cognitive and developmental tests [3-5], and decreased visual development [6]. However, preterm infants encounter a number of barriers to breastfeeding due to their immature physiological and neurodevelopmental systems. Mothers desiring to breastfeed their preterm infants are often initially encouraged to express breast milk by hand or breast pump; the expressed breast milk (EBM) is then given to the infant by gavage or bottle. Later some mothers are able to transition from gavage feeding to exclusive feeding at the breast in a short period of time without problems. Others take more extended time and experience this process as a trial and error period fraught with challenges.

In a qualitative study of mothers' experiences with breastfeeding their infants in a neonatal unit in Sweden, mothers found the process of feeding their infants at the breast to be closely regulated with strict routines including scheduled times, limits on the amount of time at the breast, and pre and post test-weighings [7]. When the mothers were successful feeding at the breast, they described feelings of pride and security. However, when their attempts were not productive, mothers expressed feelings of disappointment, frustration, rejection, shame and inadequacy, which interfered with the mother-infant relationship [7].

Upon hospital discharge, it is not unusual for mothers to continue to meet all or most of their infants' nutritional needs through bottle feedings of EBM or commercial infant formula because of their infants' weaker, less coordinated suck, problems staying alert during feedings, and difficulty in giving clear cues for hunger and satiety [8,9]. Rather than being transitioned to at-breast feedings, preterm infants are often gradually weaned from bottles of EBM to infant formula, as the mother decreases her pumping frequency leading to a diminishing supply of breast milk. In the US breastfeeding rates of preterm infants receiving mother's milk exclusively at-breast upon discharge have been found to range from 18–32% at discharge from the hospital, increasing only slightly to 23–38% by four weeks post-discharge [9,10].

The advantages of transitioning the infant to feeding at-breast may be poorly understood by some mothers and health professionals. Mothers have raised questions about at-breast feedings, such as, "Why should I try to breastfeed at the breast? Isn't the baby getting all that is needed from my pumped breast milk?" or "What are the advantages of breastfeeding at the breast as opposed to feeding bottles of breast milk? Does it really make a difference to the baby's health, growth or development?" In some cases, the mothers' inability or reluctance to transition to at-breast feedings may be due lack of knowledgeable and consistent support, teaching and assistance by the health care team. Being aware of the clear advantages of at-breast feedings may also be helpful to health care providers in providing support and information for these mothers. The purpose of this article is to discuss some of the factors that may contribute to mothers' need for support and information in transitioning to full at-breast feedings and to examine the potential short and long-term advantages associated with feeding at-breast as opposed to feeding bottles of EBM.

### Impediments toward transitioning mothers to at-breast feedings

To effectively counsel and support mothers in transitioning to full at-breast feedings, it is important for nursing and medical staff to have an understanding of the factors that may contribute to mothers' inability or resistance toward this process (Table 1). A central factor in determining the exclusivity and duration of breastfeeding for the preterm mother-infant couple is the volume of milk production. Early initiation of frequent and efficient milk expression has been found to be a key factor in adequacy of milk supply, considered by some experts to be greater than 500 ml per day [11-14]. It is likely that inadequate levels of breast milk production not only impair the infant's ability to access breast milk, but also burden the mother with "triple feeding" (feeding at the breast followed by supplementation with a bottle, then by pumping), leading mothers to abandon their efforts early.

**Table 1:** Contributing factors to mothers' inability or resistance to at-breast feedings

• Inadequate breast milk supply
• Maternal feelings of vulnerability and lack of confidence
• Infants' immature feeding behaviors
• Lack of commitment or desire to breastfeeding prior to the birth
• Personal choice
• Bottle feeding more convenient
• Ability of father or other family members to participate in feedings
• Avoidance of embarrassment of feeding in public
• Ease of pumping and storing breast milk
• Maternal lack of confidence
• Parental need to quantify intake
• Lack of informational and emotional support

Even when mothers are able to pump more than adequate milk volumes for their preterm infant, they may continue to report feelings of vulnerability related to breastfeeding in the early postpartum period [15,16]. These concerns may be promoted by the inability of some preterm infants to consume enough of the available milk because of their immature feeding behaviors. Jain and others found that preterm infants not only feed more slowly than full-term infants, but also consume lower volumes per suck [17]. This may be due to the infants' relatively low suction pressures and irregular sucking bursts [18]. Meier and Brown report that upon hospital discharge some preterm infants require complementation of breastfeeding with a bottle, supplemental nursing system, nipple shield or other breastfeeding device until the infant's gestational age reached full-term, corrected age and adequate intake of breast milk at-breast is achieved [8].

Assuming that an infant is physiologically ready for feedings at the breast, there may be more personal reasons that contribute to a mother's discomfort or reluctance toward transitioning. A mother's initial reasons for supplying breast milk during hospitalization may play a role in her decision. The choice to breastfeed or provide breast milk for a premature infant is affected by factors other than those that influence decisions of mothers of full-term infants [19]. Mothers of preterm infants may experience feelings of anxiety, vulnerability, depression and guilt surrounding the birth of the infant [20]. In response to these feelings and the information that mothers receive from nurses and physicians in the neonatal intensive care unit (NICU), some mothers change from their initial decision to formula feed to providing EBM for their infants. One of the primary extrinsic factors affecting this decision is mothers' learning of the superiority of breast milk in terms of infant growth and reduced infection rates [21]. Mothers described the offering of their breast milk to their preterm infants as a unique contribution that was likely to have a positive impact on their infants' outcome [19]. In a study of mothers of very low birth weight (VLBW) premature infants, several sociodemographic characteristics influenced breastfeeding decisions. Older White mothers who were married with higher levels of education, previous breastfeeding experience, and carried private insurance or that of a health maintenance organization were not only more likely to express breast milk initially for feedings, but also to progress to feedings at the breast [22]. Although some mothers agree to express milk with a breast pump for a short-term basis, they may have strong personal needs or reasons for not wishing to continue by feeding their infant at breast [18].

Mothers' feeding preferences for their preterm infant may be similar to those found in mothers of full-term infants, who choose to bottle feed infant formula. Bottle feeding has been reported by mothers to be more convenient, especially considering that fathers or other family members may be able to feed the infant allowing the mother more freedom in being able to leave the infant for longer periods of time [23]. The participation of the father in feeding is seen by some mothers as a good way of involving fathers in the care for the baby [24]. Bottle feeding allows some mothers to avoid the perceived embarrassment of breastfeeding in public [23]. The portability and convenience of using breast pumps may also contribute to these choices [25]. Mothers who plan on returning to work may view the pumping as needed.

Mothers' reluctance to feed the preterm infant at-breast may also be due to a maternal lack of confidence in having enough breast milk. Wooldridge and Hall studied a group of preterm infants between 30–35 weeks gestation, without any facial or gastrointestinal anomalies or identified syndromes, over a four-week period following hospital discharge [9]. They found that mothers who were able to breastfeed exclusively or feed at-breast more than half of the time had significantly higher levels of confidence than those who were giving breast milk and infant formula [9].

Another factor that may play a role in a mother's desire to continue pumping and feeding EBM is a parental need to carefully quantify the intake of her infant. In a study of mothers' concerns about breastfeeding preterm infants after discharge, the researchers reported the mothers' feelings of vulnerability were not "unreasonable, given that volume intake was measured to the nearest millimeter throughout the infants' hospital stay" [15, p30]. In response to these feelings of vulnerability, mothers may become preoccupied with schedules, times, routines and careful quantification of the infant's intake. Although several observational tools have been developed to assess effectiveness of breastfeeding, there is a lack of reliable and valid tools that can be used by mothers to visually determine milk intake for their preterm and low birth weight (LBW) infants [26]. The clinical indicators of estimating milk intake that are often used for mothers of term infants, such as changes in breast fullness after feeding and audible swallowing, have not been found to be useful in measuring adequate intake for preterm infants. A more accurate measure of milk intake at-breast has been established by pre and post test-weighings [16]. However, some clinicians are concerned that test-weighings are mechanical and unnecessary, and this concentration on numbers may interfere with breastfeeding [16,27]. The use of test-weighings varies between settings.

Finally, in some cases the inability of mothers to transition to the breast is not due to resistance on the part of the mother, but due to a lack of knowledgeable and consistent support to the mother and family. The lack of informational and emotional support by those counseling the breastfeeding mother may contribute to the reluctance of mothers to transition to breastfeeding. With shorter hospital stays in the United States, fewer preterm infants are entirely feeding at-breast prior to discharge as compared to Canada and European countries [11]. In these countries the funding of health care and cultural differences, such as extended paid maternity leave, may affect the acceptance of breastfeeding. For example, significant compensation leave benefits from employment for parents are guaranteed in Sweden after childbirth to ensure a long period of breastfeeding.

If infants are discharged prior to their full-term corrected age, it is likely that they may not be ready to fully transition to the breast. This may leave mothers in a position of finding the resources to undertake this endeavor on their own. In the US, home health agencies may have physician orders and insurance reimbursement for a few visits, but these infants are generally discharged from home care by one to two weeks post hospital discharge. Further, the expertise needed in helping a mother transition to the breast often requires an expert in lactation. In a case study of transitioning a 36-week-old premature infant to the breast after hospital discharge, Drosten describes the difficulty and time involved for a mother, who had a routine of pumping six times per day as well as attempting to slowly breastfeed her infant before every bottle feeding [28]. She gives recommendations that included special positioning of the infant for feedings, using breastfeeding devices for supplementation, developing a realistic but adequate pumping schedule with an increased frequency of up to eight times a day, and monitoring feeding schedules and weight gains. Once an infant is discharged from the hospital, these services may be unavailable to mothers who cannot afford a private lactation consultant in the US.

### Benefits of at-breast feedings

Although the advantages of breastfeeding have been well described in the literature, the focus of this section is on the specific advantages of feeding at the breast as opposed to bottle feedings of EBM. Mothers, who are expressing breast milk for their preterm infants and feeding it by bottle, might benefit from the knowledge of the advantages of feeding at the breast in order to make the decision whether or not to move to the next level of breastfeeding. Feeding an infant at the breast has been found to provide physiological benefits to the infant, as well as physical, psychological and pragmatic benefits to the mother (Table 2). Some of these advantages appear to be short-term, whereas others become more evident over longer periods of time.

**Table 2:** Benefits of feeding at-breast as opposed to bottle feeding expressed breast milk (EBM)

**Benefits to infant**	
Improved oxygenation and temperature regulation during feedings	• Higher oxygen saturation • Better coordinated sucking, swallowing, breathing pattern • Increased body temperature • Fewer episodes of apnea and bradycardia
Advantages of skin-to-skin contact	• Increased breast milk volume • Greater production of maternal milk antibodies to pathogens in infant's environment
Enhanced nutritional and immunological properties of breast milk	• Superior nutritional content lost by freezing, thawing and reheating EBM • Lower risk of bacterial contamination and growth due to handling
Better oral development	• Optimal mandibular development • Strengthening of the jaw muscles • Increased nasal cavity space • Improved future teeth alignment and decrease in malocclusions • Greater breathing efficiency
More efficient emptying of the breast	• Greater milk volume in same amount of time as breast pump • Increase in milk volume over time

**Benefits to mother**	

Reduced risk of breast trauma	• Less risk of mastitis with ineffective emptying of breast • Lower risk of damage to nipple from breast pump
Reduced risks to mothers' health	• Decreased incidence of type 2 diabetes • Reduced risk of breast cancer
Psychological effects	• Potential reduction in perceived stress and negative mood after feedings
Practical advantages	• Less time in preparing EBM for feeding and cleaning of supplies • Breast milk at optimal temperature without preparation • Cost savings in not renting or buying an electric breast pump

#### Physiological benefits for infants

The primary short-term benefits of an infant receiving breast milk at the breast are physiological. In studies conducted by Chen and others, the researchers found that in comparison to bottle-feeding events, preterm infants tend to have higher oxygen saturations when they were directly breastfed, which may be due to more coordinated sucking, swallowing and breathing during breastfeeding [29]. They also found the breastfed infants' body temperatures were higher during feedings despite the bottle feeding infants being fed inside the incubator. The researchers propose that this finding may be related to the infants' position during breastfeeding, the skin-to-skin contact with the mother, and the temperature of the breast milk. Preterm infants have also been found to have less variation from baseline in heart rate, respiratory rate and oxygen saturation, and fewer episodes of apnea and bradycardia during breastfeeding when compared to bottle-feeding [30-32]. These findings may be due to infants' ability to breathe easier during sucking bursts and to regulate their breathing pattern during sucking pauses while breastfeeding. Breast milk not only helps the infant nutritionally, but taken at-breast appears to benefit the infant physiologically.

#### Skin-to-skin contact

Feeding an infant at-breast necessitates some skin-to-skin contact between the infant and mother. Mothers who hold their premature infants skin-to-skin (also known as kangaroo care) often experience an increase in breast milk volume and possibly a greater production of maternal milk antibodies to specific pathogens in the infant's surroundings [33]. Therefore, it is feasible that some of the benefits experienced with skin-to-skin care may also emerge through breastfeeding at the breast.

#### Nutritional & immunological benefits

Taking breast milk at the breast rather than from a bottle offers other advantages to the infant. Breast milk loses some of its nutritional and immunological properties by freezing, thawing and reheating in the process of expressing the breast milk prior to feeding it with a bottle, which reduces the protective benefits offered by breast milk [18]. Rapid heating, especially in a microwave oven, and at high temperatures contributes to an even greater loss of beneficial components. For example, ascorbic acid levels decrease significantly when stored at low temperatures, and have been found to drop 40% when reheating [34]. Further, there may be risks of bacterial contamination and growth, if the breast milk is not expressed and handled appropriately.

#### Infant oral developmental effects

In addition to the potential physiological, nutritional, immunological and more efficient advantages offered by receiving breast milk at-breast, infants may benefit from fewer dental problems later in life. According to Palmer, breastfeeding at-breast has a positive effect on the development of an infant's oral cavity including optimal mandibular development, strengthening of the jaw muscles, and increased nasal cavity space [35]. During breastfeeding, the tongue, lower lip and mandible move in concert to draw the milk into the mouth by a stripping action, gently shaping the infant's hard palate. This process leads to an enhanced formation of the hard palate producing improved future teeth alignment and a decrease in malocclusions. Whereas the shape of the breast-nipple is in a geometric form consistent with the infant's mouth, the artificial nipple in bottle-feeding hinders the formation of the jaw muscles. Palmer also proposes that the increase in nasal space may have a significant effect on the person's breathing efficiency, reducing later problems with snoring and obstructive sleep apnea [35].

#### Efficiency of feeding

Another potential advantage of feeding at the breast for infants who have reached term is related to the infant's efficiency of feeding. Zoppou, Barry and Mercer used a computer model to compare the differences between breastfeeding and breast pumps [36]. The pump relies on suction to remove milk, whereas the infant uses a massaging motion of the tongue and jaw to apply a peristaltic force through compression of the nipple and most of the areola at a particular time during the suction cycle. This peristaltic force acts as a stripping mechanism of the milk from the breast. The amount and timing of the peristaltic force is crucial in increasing the amount of milk volume obtained. In comparison to breast pumps that only apply suction, the researchers were able to demonstrate an increase of 15% in milk volume, when they altered the speed of the peristaltic force and the time it was utilized in the suction cycle. The data suggest that once infants reach term and have an appropriate strong, coordinated suck, they may get more milk volume in the same amount of time as a breast pump, and that the suction and compression seen in breastfeeding are mutually dependent during suckling. Hill and others recently compared the milk output of mothers of preterm and term infants [37]. They found that whereas the milk output increased for the mothers of term infants over time, it remained stable or declined for the mothers of the preterm infants, who were using regular mechanical expression [37].

#### Reduced risk of breast trauma

Although electric breast pumps have been crucial for the milk supply of many mothers of preterm infants, they also offer some potential disadvantages. Problems with breast pumps may be overlooked and contribute to ineffective emptying of the breast, especially if they are underpowered or poor fitting [38]. Some researchers have suggested that the breast pump's reduced physiologic mechanism may negatively affect emptying, especially in the periphery of the breast and may be a contributing factor to the association between pump use and mastitis [39]. Another disadvantage of breast pumps is that they work primarily on suction, and may be ineffective if set at too low a level (<150 mmHg) or cause damage to the nipple skin if set too high (>200 mmHg) [36].

#### Lack of breastfeeding is a risk to mothers' health

It is reasonable that when mothers are successful breastfeeding at the breast, they are more likely to continue breastfeeding longer, and reduce the health risks of not breastfeeding. Breastfeeding reduces mothers' risk of developing type 2 diabetes later in life [40]. In an analysis of data from 47 epidemiological studies in 30 countries, breastfeeding was also found to reduce the relative risk of breast cancer by 4.3% for every year of a woman's life spent breastfeeding [41].

#### Psychological effects of breastfeeding

The act of breastfeeding has also been associated with positive psychological effects for the mother in respect to mood and stress. Mezzacappa and Katkin conducted a study among 28 mothers who were both breastfeeding and bottle feeding [42]. After a breastfeeding session, the mothers were found to have a reduction in perceived stress and negative mood relative to what was found after a bottle feeding. In contrast, bottle feeding appeared to decrease positive mood. The researchers suggested that the higher levels of the hormone oxytocin released by breastfeeding contributed to the decrease in negative mood [42]. The authors also speculated that over time breastfeeding may condition the mother to regard her infant positively leading to greater mother-infant attachment, and the decrease in negative mood and stress associated with breastfeeding may reduce the risk postpartum depression [42]. These findings are consistent with those found by Shepherd and others, who reported a higher maternal contentment level and a positive emotional experience in women who breastfed their infants [24].

#### Practical advantages for mothers

Finally, there are the practical advantages for a mother, who transitions to at-breast feedings rather than continuing to feed EBM. One benefit is the savings in time and effort of feeding. Hours of the mothers' time, which were previously spent in expressing breast milk and later preparing it for refeeding and cleaning bottles and breast pump equipment, are saved. The milk is always available in the breast at an ideal temperature. The time and effort spent in pumping and refeeding around the clock has clear advantages for the infant with immature sucking skills, but once the infant is able to efficiently feed at-breast, these efforts may become overwhelming for the mother leading to early weaning to infant formula. There may also be a considerable cost savings to the mother in not having to rent or buy an electric breast pump.

### Breastfeeding advice falling short

Some neonatal units are more successful than others in transitioning infants to at-breast feedings prior to discharge. In a study of interventions for VLBW infants, Miracle and others reported that one of the primary reasons for the high rates of lactation in the unit they studied was due to the encouragement the mothers received from the NICU nurses and physicians [19]. Mothers wanted the care providers to discuss the benefits of human milk feeding in order that they could make an informed decision [19].

Even in NICUs where mothers are encouraged by health care professionals to provide breast milk to their preterm infants, is the advice falling short? Hurst and Meier suggest that mothers who did not intend prenatally to breastfeed their preterm infants may be reluctant to begin breast milk expression for their infant in the NICU if they have to make a commitment for several months [18]. Further, they recommend that health care professionals should not encourage mothers to feel that an alternative to long-term exclusive breastfeeding is "second best." While the message in the NICU may be to get the baby off to the "best start" and to postpone other breastfeeding decisions, there are potential risks of not providing mothers prior to hospital discharge with the opportunity and clear benefits of eventually transitioning to feeding at-breast as opposed to only pumping and feeding EBM.

One of the primary factors contributing to early termination of breastfeeding in mothers of preterm infants is the failure to transition from breast milk to at-breast feedings. In a study of mothers of VLBW infants, the mothers who lactated the longest were those who were expressing at least five times a day and had put the infant to breast by 35 weeks corrected age [12]. In another study of low-birth-weight (LBW) infants in a Swedish neonatal unit, mothers were encouraged to initiate breastfeeding as soon as their infants were clinically stable rather than a standard based on weight or gestational age [11]. This practice combined with progressing from tube to breastfeeding and extended mother-infant exposure through kangaroo care resulted in 93% of the 70 LBW infants receiving their mothers' milk at discharge, and 95% of them at the breast [11]. Although the decision to transition the infant to at-breast feedings may not carry the same "life and death" risks as compared to feeding a preterm infant EBM at earlier stages of development, the extended immune protection of breastfeeding could have a major impact on the length of time the infant receives any breast milk as well as the infant's overall health and development.

### Implications for education and practice

Health care providers should have a sound understanding of the factors that impede mothers from transitioning to at-breast feedings in order to address mothers' primary concerns and to keep their perspectives in mind during discussions about feedings. For example, if a mother's major concern is the need to quantify intake, this could be addressed with assisting her in obtaining an electronic scale for infant pre and post test-weighings, and giving clear parameters on how to transition to the breast. Although the message should not contain value statements about the right or wrong way of feeding infants, mothers need factual evidence-based information about the nature of maternal concerns regarding transitioning to the breast and the potential returns of at-breast feedings in order to make an informed choice.

## Conclusion

Preterm infants who receive expressed breast milk rather than formula have fewer infections and necrotizing enterocolitis, as well as better cognitive, neurological and visual development. However, an optimal outcome would be for the preterm infant to transition to at-breast feedings prior to or shortly after hospital discharge. But with earlier discharges and limited home health care follow-up, mothers may not receive the informational and emotional support to transition to the breast. Health professionals and mothers may benefit from a clear understanding of the factors that impede mothers of preterm infants from transitioning to at-breast feedings, and their potential outcomes.

## Competing interests

The author(s) declare that they have no competing interests.
